# COVID-19: сonsequences for mental health and the use of e-Mental health options

**DOI:** 10.17650/2712-7672-2020-1-1-3-7

**Published:** 2020-09-02

**Authors:** Wolfgang Gaebel, Rabea Lukies, Johannes Stricker

**Affiliations:** Department of Psychiatry, Medical Faculty, LVR-Klinikum Düsseldorf, Heinrich-Heine-University; WHO Collaborating Centre for Quality Assurance and Empowerment in Mental Health; LVR-Institute for Healthcare Research

**Keywords:** e-mental health, mental health, COVID-19, pandemic, электронные технологии охраны психического здоровья, психическое здоровье, COVID-19, пандемия

## Abstract

The current COVID-19 pandemic is associated with fear, insecurity, and perceived social isolation worldwide. In this editorial, we discuss the influence of the COVID-19 pandemic on mental health among the general population and among particularly vulnerable groups (e.g., people with pre-existing mental illness). Additionally, we explore the role of e-mental health options in times of social distancing. Preliminary empirical evidence indicates that a wide range of people have experienced mental health difficulties due to the COVID-19 pandemic and corresponding infection-control measures. E-mental health options are a feasible means of addressing psychological distress and mental illness during the pandemic. Thus, these options should be made available in a timely fashion. Future multidisciplinary research is needed to develop e-mental health strategies that specifically focus on the consequences of social isolation, economic hardship and fear of infection.

## INTRODUCTION

The current COVID-19 pandemic has caused insecurity and fear around the globe. Additionally, measures such as social distancing and home confinement, which help prevent the spread of the disease, carry the risk of increasing psychological distress or aggravation of preexisting mental illness. Thus, mental health experts worldwide have raised concerns over the consequences of the COVID-19 pandemic for mental health (e.g., [1–4]). These concerns are in line with previous evidence relating to mental health problems following isolation measures in the context of contagious diseases [5]. There is also some preliminary evidence indicating the negative effects of the current COVID-19 pandemic on mental health with stricter and more efficient social distancing measures (e.g., quarantine), potentially causing more severe consequences [2, 6].

The World Health Organization (WHO), the Inter-Agency Standing Committee (IASC) created by the United Nations (UN), the World Psychiatric Association (WPA), and several other organizations have published guidelines and resources for maintaining or restoring mental health during the pandemic [7–9]. Additionally, national organizations are setting up tailored national strategies to respond to mental health issues during the pandemic. Many of these strategies include online-based mental health options (e.g., [1, 10, 11]), which is partly due to difficulties associated with inpatient mental health services (e.g., the risk of infections; [12]). In this editorial, we discuss evidence for the impact of the current COVID-19 pandemic on mental health among the general population and among vulnerable groups (e.g., persons with pre-existing mental illness). Additionally, we explore how the use of e-mental health options can help maintain or restore mental health in the current COVID-19 pandemic.

## CONSEQUENCES OF THE COVID-19 PANDEMIC FOR MENTAL HEALTH

The COVID-19 pandemic may negatively influence mental health via increased stress levels due to fears about infection, economic hardship, job loss or via perceived social isolation as a consequence of social distancing measures. Additionally, excessive fear and panic due to misinformation on social media (the so-called "infodemic") may occur [13], which has been termed "coronaphobia" [14]. According to a recent review based on 28 primary articles (including four empirical studies), anxiety and depressive symptoms have been common during the COVID-19 pandemic among the general population (16-28% point prevalence; [15]). There is also some anecdotal evidence of suicides because of fears related to the COVID-19 pandemic [16]. Similarly, anecdotal evidence from Germany indicates the occurrence of specific symptom clusters, such as a nihilistic "apocalyptic" syndrome among elderly patients who have been cut off from their families during the pandemic [17].

Some societal groups may be particularly vulnerable to the psychological consequences of the COVID-19 pandemic. As stress increases the risk of relapse or aggravation of pre-existing mental illness, persons with pre-existing mental illness may be particularly prone to experiencing difficulties [12, 18]. Additionally, persons with mental illness may avoid inpatient or outpatient services due to fear of infection during the pandemic. Similarly, infection-control measures (e.g., travel restrictions) may complicate regular outpatient visits. Other potentially vulnerable groups include healthcare workers (e.g., [3]), older persons (e.g., [19]), students studying overseas (e.g., [20]), homeless persons [21] and migrant workers [22]. For example, in a recent study of Chinese medical staff during the COVID-19 outbreak, 50.7% of the participating medical staff reported depressive symptoms; 44.7% reported anxiety symptoms; 36.1% reported symptoms of insomnia and 73.4% reported general stress-related symptoms [23].

For certain groups, living circumstances can also influence the degree to which persons experience mental health difficulties during the COVID-19 pandemic. In a study of college students in China [24] who were living in urban areas (as opposed to rural areas), the stability of a student's family income and living with parents were found to be protective factors against anxiety during the pandemic. Results indicated that stressors related to anxiety levels included worry about economic influences, worry about academic delays and the influence on daily life, whereas social support could have a buffering effect [24]. An innovative study applying machine learning algorithms to postings on social media found that social media users in China more frequently posted about negative emotions (e.g., anxiety, depression and indignation) after the COVID-19 outbreak, compared to before [25].

In sum, there is preliminary evidence of substantial negative effects of the COVID-19 pandemic on mental health. As more people have been affected by COVID-19 compared to previous pandemics (e.g., SARS or H1N1 influenza), the challenges for mental healthcare seem unprecedentedly large. Against this background, with such severe consequences resulting from the COVID-19 pandemic in terms of mental health, it becomes clear that sustainable solutions are needed to address psychological distress and mental illness in the current situation.

## THE USE OF E-MENTAL HEALTH OPTIONS IN THE COVID-19 PANDEMIC

Various stakeholders have recommended the use of e-mental health options as a means of addressing the negative impacts of the COVID-19 pandemic on mental health (e.g., [4, 26]). Some of these recommendations cover mental health options that are adequate for persons with and without pre-existing mental illness. Such options include, for example, the use of online materials for education in mental health (e.g., [23]) or applications covering self-help (e.g., [27]). Other recommendations, such as synchronous telemedicine in mental healthcare for diagnosis and counselling (e.g., [26]) and asynchronous therapeutic intervention such as structured letter therapy (e.g., [28]), are tailored for persons with mental illness. Growing evidence shows the general effectiveness of digital solutions for improving mental health (e.g., [29, 30]). A recent review also showed that e-mental health interventions may be effective in crisis settings [31].

The COVID-19 pandemic may be a "turning point" for e-mental health, which may durably increase the uptake of e-mental health options [27]. In China (the first country to be affected by the pandemic), online mental health options (e.g., online mental health education, online psychological counselling and online psychological self-help intervention systems services) have been widely used during the pandemic [23]. However, to date, many countries are not yet in a position to support the use of e-mental health solutions during the pandemic. In Germany, just this year, the Digital Health Care Act has come into effect, enabling the prescription of medical apps. A consistent digital infrastructure throughout healthcare institutions is still lacking. Recently, however, in response to the COVID-19 outbreak, the National Association of Statutory Health Insurance Physicians and the Association of Statutory Health Insurances have agreed to expand the reimbursement possibilities for video consultations [32]. Thus, the pandemic seems to have accelerated the regulatory processes required for the use of e-mental health services.

National governments, health policymakers and crisis policies should consider further accelerating the implementation of e-mental health in order to prevent or reduce psychological distress among the general population and to restore mental health in persons with pre-existing mental illness. As a quick emergency response, governments could expand the legal framework that allows the application and reimbursement of e-mental health products and publish guidance for their use. However, for an increased uptake in the long run, more sustainable policy measures must be introduced, including development of adequate financing strategies, the funding of further research, promotion of high standards of usability and interoperability, and quality control for e-mental health products on the market [33, 34].

In sum, e-mental health options should quickly be made available to all persons potentially suffering from the psychological consequences of the COVID-19 pandemic, particularly to vulnerable groups, such as persons with pre-existing mental illness, healthcare professionals and older people. Future multidisciplinary research is needed to develop e-mental health strategies that specifically focus on the consequences of social isolation, economic hardship and fear of infection (see also [35]).

## Author contributions 

Rabea Lukies and Johannes Strieker contributed equally to researching literature and writing the manuscript. Prof. Dr. Wolfgang Gaebel provided ideas about content and literature, and critically revised the draft manuscript.

## Funding 

Prof. Dr. Wolfgang Gaebel and Rabea Lukies are part of the eMEN project cited in the manuscript, which received funding from the European Union’s Interreg NorthWest-Europe programme.

## Conflict of interest

The authors declare that they have no conflicts of interest.
